# Supplementary material for: Local cortical desynchronization and pupil-linked arousal differentially shape brain states for optimal sensory performance
Source: eLife. 2019 Dec 10;8:e51501. doi: 10.7554/eLife.51501 (PMC6946578; doi:10.7554/eLife.51501)
Supplement: Supplementary file 1. — The table shows model coefficients, standard errors, effect size estimates as well as goodness of fit statistics for the model reported in results and discussion sections. [file elife-51501-supp1.docx]

| **Table S1: Brain-brain model predicting pre-stimulus low-frequency power** | | | | | |
| --- | --- | --- | --- | --- | --- |
|  | **Pre-stimulus low frequency power** | | | | |
| *Predictors* | *Estimates* | *std. Error* | *CI* | *t-value* | *p* |
| Intercept | -0.041 | 0.038 | -0.116 – 0.033 | -1.089 | 0.2760 |
| **Entropy (linear)** | **-0.179** | **0.011** | **-0.200 – -0.158** | **-16.570** | **<0.001** |
| **Entropy (quadratic)** | **0.030** | **0.009** | **0.012 – 0.048** | **3.299** | **0.0010** |
| Entropy baseline | 0.045 | 0.013 | 0.020 – 0.070 | 3.519 | 0.0004 |
| **Pupil size (linear)** | **-0.041** | **0.010** | **-0.062 – -0.021** | **-3.923** | **0.0001** |
| **Pupil size (quadratic)** | **0.016** | **0.006** | **0.003 – 0.028** | **2.504** | **0.0123** |
| Entropy (linear) x Baseline | -0.000 | 0.001 | -0.003 – 0.003 | -0.071 | 0.9434 |
| Entropy(quadratic) x Baseline | -0.013 | 0.010 | -0.033 – 0.007 | -1.249 | 0.2115 |
| Participant | 0.007 | 0.007 | -0.006 – 0.020 | 1.014 | 0.3106 |
| Observations | 9831 | | | | |
| R^2^ / adjusted R^2^ | 0.033 / 0.032 | | | | |

**Supplementary file 1. Estimates and statistics of the model predicting pre-stimulus low-frequency power.**
