# Supplementary material for: Local cortical desynchronization and pupil-linked arousal differentially shape brain states for optimal sensory performance
Source: eLife. 2019 Dec 10;8:e51501. doi: 10.7554/eLife.51501 (PMC6946578; doi:10.7554/eLife.51501)
Supplement: Supplementary file 2. — The table shows model coefficients, standard errors, effect size estimates as well as goodness of fit statistics for the model reported in results and discussion sections. [file elife-51501-supp2.docx]

| **Table S2: Brain-brain model predicting pre-stimulus alpha power** | | | | | |
| --- | --- | --- | --- | --- | --- |
|  | **Pre-stimulus alpha power** | | | | |
| *Predictors* | *Estimates* | *std. Error* | *CI* | *t-value* | *p* |
| Intercept | -0.047 | 0.037 | -0.120 – 0.026 | -1.268 | 0.2049 |
| **Entropy (linear)** | **-0.291** | **0.011** | **-0.311 – -0.270** | **-27.575** | **<0.001** |
| **Entropy (quadratic)** | **0.057** | **0.009** | **0.039 – 0.074** | **6.372** | **<0.001** |
| Entropy baseline | 0.077 | 0.012 | 0.052 – 0.101 | 6.204 | <0.001 |
| Pupil size (linear) | 0.009 | 0.010 | -0.010 – 0.029 | 0.930 | 0.3524 |
| Pupil size (quadratic) | -0.004 | 0.006 | -0.016 – 0.009 | -0.566 | 0.5715 |
| Entropy (linear) x Baseline | -0.000 | 0.001 | -0.003 – 0.003 | -0.097 | 0.9230 |
| Entropy(quadratic) x Baseline | -0.019 | 0.010 | -0.038 – 0.001 | -1.880 | 0.0601 |
| Participant | 0.011 | 0.007 | -0.002 – 0.024 | 1.651 | 0.0988 |
| Observations | 9831 | | | | |
| R^2^ / adjusted R^2^ | 0.082 / 0.082 | | | | |

**Supplementary file 2. Estimates and statistics of the model predicting pre-stimulus alpha power.**
