# Supplementary material for: Local cortical desynchronization and pupil-linked arousal differentially shape brain states for optimal sensory performance
Source: eLife. 2019 Dec 10;8:e51501. doi: 10.7554/eLife.51501 (PMC6946578; doi:10.7554/eLife.51501)
Supplement: Supplementary file 3. — The table shows model coefficients, standard errors, effect size estimates as well as goodness of fit statistics for the model reported in results and discussion sections. [file elife-51501-supp3.docx]

| **Table S3: Brain-brain model predicting pre-stimulus beta power** | | | | | |
| --- | --- | --- | --- | --- | --- |
|  | **Pre-stimulus beta power** | | | | |
| *Predictors* | *Estimates* | *std. Error* | *CI* | *t-value* | *p* |
| Intercept | -0.062 | 0.037 | -0.134 – 0.010 | -1.699 | 0.0893 |
| **Entropy (linear)** | **-0.316** | **0.010** | **-0.336 – -0.296** | **-30.329** | **<0.001** |
| **Entropy (quadratic)** | **0.074** | **0.009** | **0.056 – 0.091** | **8.336** | **<0.001** |
| Entropy baseline | 0.106 | 0.012 | 0.082 – 0.130 | 8.701 | <0.001 |
| **Pupil size (linear)** | **0.043** | **0.010** | **0.023 – 0.063** | **4.267** | **<0.001** |
| Pupil size (quadratic) | -0.004 | 0.006 | -0.016 – 0.008 | -0.582 | 0.5608 |
| Entropy (linear) x Baseline | -0.000 | 0.001 | -0.003 – 0.002 | -0.106 | 0.9158 |
| Entropy(quadratic) x Baseline | -0.026 | 0.010 | -0.045 – -0.006 | -2.603 | 0.0093 |
| Participant | 0.014 | 0.007 | 0.001 – 0.027 | 2.128 | 0.0333 |
| Observations | 9831 | | | | |
| R^2^ / adjusted R^2^ | 0.102 / 0.102 | | | | |

**Supplementary file 3. Estimates and statistics of the model predicting pre-stimulus beta-power.**
