# Supplementary material for: Local cortical desynchronization and pupil-linked arousal differentially shape brain states for optimal sensory performance
Source: eLife. 2019 Dec 10;8:e51501. doi: 10.7554/eLife.51501 (PMC6946578; doi:10.7554/eLife.51501)
Supplement: Supplementary file 4. — The table shows model coefficients, standard errors, effect size estimates as well as goodness of fit statistics for the model reported in results and discussion sections. [file elife-51501-supp4.docx]

| **Table S4: Brain-brain model predicting pre-stimulus gamma power** | | | | | |
| --- | --- | --- | --- | --- | --- |
|  | **Pre-stimulus gamma power** | | | | |
| *Predictors* | *Estimates* | *std. Error* | *CI* | *t-value* | *p* |
| Intercept | -0.054 | 0.038 | -0.129 – 0.020 | -1.426 | 0.1539 |
| **Entropy (linear)** | **-0.179** | **0.011** | **-0.200 – -0.157** | **-16.543** | **<0.001** |
| **Entropy (quadratic)** | **0.061** | **0.009** | **0.043 – 0.079** | **6.655** | **<0.001** |
| Entropy baseline | 0.049 | 0.013 | 0.024 – 0.074 | 3.842 | 0.0001 |
| Pupil size (linear) | -0.013 | 0.010 | -0.034 – 0.007 | -1.275 | 0.2022 |
| Pupil size (quadratic) | 0.001 | 0.006 | -0.012 – 0.013 | 0.127 | 0.8992 |
| Entropy (linear) x Baseline | -0.000 | 0.001 | -0.003 – 0.003 | -0.066 | 0.9474 |
| Entropy(quadratic) x Baseline | -0.022 | 0.010 | -0.042 – -0.002 | -2.178 | 0.0294 |
| Participant | 0.001 | 0.007 | -0.012 – 0.014 | 0.148 | 0.8822 |
| Observations | 9831 | | | | |
| R^2^ / adjusted R^2^ | 0.035 / 0.034 | | | | |

**Supplementary file 4. Estimates and statistics of the model predicting pre-stimulus gamma power.**
