# Supplementary material for: Local cortical desynchronization and pupil-linked arousal differentially shape brain states for optimal sensory performance
Source: eLife. 2019 Dec 10;8:e51501. doi: 10.7554/eLife.51501 (PMC6946578; doi:10.7554/eLife.51501)
Supplement: Supplementary file 5. — The table shows model coefficients, standard errors, effect size estimates as well as goodness of fit statistics for the model reported in results and discussion sections. [file elife-51501-supp5.docx]

| **Table S5: Brain-brain model predicting post-stimulus low-frequency power** | | | | | |
| --- | --- | --- | --- | --- | --- |
|  | **Stimulus-evoked low frequency power** | | | | |
| *Predictors* | *Estimates* | *std. Error* | *CI* | *t-value* | *p* |
| Intercept | 0.001 | 0.039 | -0.074 – 0.077 | 0.038 | 0.9694 |
| **Entropy (linear)** | **-0.026** | **0.011** | **-0.048 – -0.005** | **-2.387** | **0.0170** |
| Entropy (quadratic) | 0.001 | 0.009 | -0.017 – 0.019 | 0.107 | 0.9149 |
| Entropy baseline | 0.043 | 0.013 | 0.018 – 0.069 | 3.376 | 0.0007 |
| Pupil size (linear) | 0.015 | 0.011 | -0.006 – 0.035 | 1.369 | 0.1711 |
| Pupil size (quadratic) | 0.010 | 0.006 | -0.002 – 0.023 | 1.604 | 0.1088 |
| Entropy (linear) x Baseline | -0.000 | 0.001 | -0.003 – 0.003 | -0.159 | 0.8740 |
| Entropy(quadratic) x Baseline | -0.038 | 0.010 | -0.058 – -0.018 | -3.660 | 0.0003 |
| Participant | 0.014 | 0.007 | 0.000 – 0.027 | 2.000 | 0.0456 |
| Observations | 9831 | | | | |
| R^2^ / adjusted R^2^ | 0.006 / 0.005 | | | | |

**Supplementary file 5. Estimates and statistics of the model predicting post-stimulus low-frequency power.**
