# Supplementary material for: Local cortical desynchronization and pupil-linked arousal differentially shape brain states for optimal sensory performance
Source: eLife. 2019 Dec 10;8:e51501. doi: 10.7554/eLife.51501 (PMC6946578; doi:10.7554/eLife.51501)
Supplement: Supplementary file 6. — The table shows model coefficients, standard errors, effect size estimates as well as goodness of fit statistics for the model reported in results and discussion sections. [file elife-51501-supp6.docx]

| **Table S6: Brain-brain model predicting post-stimulus alpha power** | | | | | |
| --- | --- | --- | --- | --- | --- |
|  | **Post-stimulus alpha power** | | | | |
| *Predictors* | *Estimates* | *std. Error* | *CI* | *t-value* | *p* |
| Intercept | 0.012 | 0.039 | -0.064 – 0.088 | 0.311 | 0.7558 |
| Entropy (linear) | -0.008 | 0.011 | -0.029 – 0.014 | -0.706 | 0.4801 |
| Entropy (quadratic) | -0.011 | 0.009 | -0.029 – 0.007 | -1.160 | 0.2461 |
| Entropy baseline | 0.043 | 0.013 | 0.018 – 0.069 | 3.374 | 0.0007 |
| **Pupil size (linear)** | **0.033** | **0.011** | **0.013 – 0.054** | **3.141** | **0.0017** |
| Pupil size (quadratic) | -0.000 | 0.006 | -0.013 – 0.012 | -0.027 | 0.9785 |
| Entropy (linear) x Baseline | -0.000 | 0.001 | -0.003 – 0.003 | -0.042 | 0.9669 |
| Entropy(quadratic) x Baseline | -0.002 | 0.010 | -0.022 – 0.019 | -0.145 | 0.8847 |
| Participant | 0.007 | 0.007 | -0.007 – 0.020 | 0.947 | 0.3438 |
| Observations | 9831 | | | | |
| R^2^ / adjusted R^2^ | 0.004 / 0.003 | | | | |

**Supplementary file 6. Estimates and statistics of the model predicting post-stimulus alpha power.**
