# Supplementary material for: Local cortical desynchronization and pupil-linked arousal differentially shape brain states for optimal sensory performance
Source: eLife. 2019 Dec 10;8:e51501. doi: 10.7554/eLife.51501 (PMC6946578; doi:10.7554/eLife.51501)
Supplement: Supplementary file 7. — The table shows model coefficients, standard errors, effect size estimates as well as goodness of fit statistics for the model reported in results and discussion sections. [file elife-51501-supp7.docx]

| **Table S7: Brain-brain model predicting post-stimulus beta power** | | | | | |
| --- | --- | --- | --- | --- | --- |
|  | **Post-stimulus beta power** | | | | |
| *Predictors* | *Estimates* | *std. Error* | *CI* | *t-value* | *p* |
| Intercept | -0.014 | 0.039 | -0.089 – 0.062 | -0.349 | 0.7268 |
| Entropy (linear) | -0.006 | 0.011 | -0.027 – 0.016 | -0.516 | 0.6059 |
| Entropy (quadratic) | 0.001 | 0.009 | -0.017 – 0.020 | 0.150 | 0.8806 |
| Entropy baseline | 0.007 | 0.013 | -0.019 – 0.032 | 0.521 | 0.6026 |
| Pupil size (linear) | 0.007 | 0.011 | -0.014 – 0.028 | 0.656 | 0.5122 |
| Pupil size (quadratic) | 0.008 | 0.006 | -0.004 – 0.021 | 1.292 | 0.1963 |
| Entropy (linear) x Baseline | 0.000 | 0.001 | -0.003 – 0.003 | 0.026 | 0.9790 |
| Entropy(quadratic) x Baseline | 0.012 | 0.010 | -0.009 – 0.032 | 1.128 | 0.2594 |
| Participant | 0.002 | 0.007 | -0.012 – 0.015 | 0.225 | 0.8218 |
| Observations | 9831 | | | | |
| R^2^ / adjusted R^2^ | 0.001 / -0.000 | | | | |

**Supplementary file 7. Estimates and statistics of the model predicting post-stimulus beta power.**
