# Supplementary material for: Local cortical desynchronization and pupil-linked arousal differentially shape brain states for optimal sensory performance
Source: eLife. 2019 Dec 10;8:e51501. doi: 10.7554/eLife.51501 (PMC6946578; doi:10.7554/eLife.51501)
Supplement: Supplementary file 8. — The table shows model coefficients, standard errors, effect size estimates as well as goodness of fit statistics for the model reported in results and discussion sections. [file elife-51501-supp8.docx]

| **Table S8: Brain-brain model predicting post-stimulus gamma power** | | | | | |
| --- | --- | --- | --- | --- | --- |
|  | **Post-stimulus gamma power** | | | | |
| *Predictors* | *Estimates* | *std. Error* | *CI* | *t-value* | *p* |
| Intercept | 0.012 | 0.039 | -0.064 – 0.088 | 0.313 | 0.7546 |
| **Entropy (linear)** | **0.040** | **0.011** | **0.018 – 0.061** | **3.598** | **0.0003** |
| Entropy (quadratic) | -0.007 | 0.009 | -0.026 – 0.011 | -0.783 | 0.4337 |
| Entropy baseline | 0.013 | 0.013 | -0.012 – 0.038 | 1.021 | 0.3074 |
| Pupil size (linear) | -0.016 | 0.011 | -0.037 – 0.004 | -1.539 | 0.1239 |
| Pupil size (quadratic) | 0.004 | 0.006 | -0.008 – 0.017 | 0.671 | 0.5023 |
| Entropy (linear) x Baseline | -0.000 | 0.001 | -0.003 – 0.003 | -0.069 | 0.9449 |
| Entropy(quadratic) x Baseline | -0.029 | 0.010 | -0.050 – -0.009 | -2.798 | 0.0051 |
| Participant | -0.003 | 0.007 | -0.017 – 0.010 | -0.458 | 0.6468 |
| Observations | 9831 | | | | |
| R^2^ / adjusted R^2^ | 0.003 / 0.002 | | | | |

**Supplementary file 8. Estimates and statistics of the model predicting post-stimulus gamma power.**
