# Supplementary material for: Local cortical desynchronization and pupil-linked arousal differentially shape brain states for optimal sensory performance
Source: eLife. 2019 Dec 10;8:e51501. doi: 10.7554/eLife.51501 (PMC6946578; doi:10.7554/eLife.51501)
Supplement: Supplementary file 9. — The table shows model coefficients, standard errors, effect size estimates as well as goodness of fit statistics for the model reported in results and discussion sections. [file elife-51501-supp9.docx]

| **Table S9: Brain-brain model predicting post-stimulus ITC** | | | | | |
| --- | --- | --- | --- | --- | --- |
|  | **Post-stimulus low frequency ITC** | | | | |
| *Predictors* | *Estimates* | *std. Error* | *CI* | *t-value* | *p* |
| Intercept | 0.035 | 0.039 | -0.041 – 0.110 | 0.897 | 0.3697 |
| **Entropy (linear)** | **0.052** | **0.011** | **0.031 – 0.074** | **4.759** | **<0.001** |
| **Entropy (quadratic)** | **-0.022** | **0.009** | **-0.040 – -0.004** | **-2.361** | **0.0182** |
| Entropy baseline | -0.016 | 0.013 | -0.042 – 0.009 | -1.276 | 0.2021 |
| Pupil size (linear) | 0.002 | 0.011 | -0.018 – 0.023 | 0.227 | 0.8207 |
| Pupil size (quadratic) | -0.005 | 0.006 | -0.017 – 0.008 | -0.720 | 0.4715 |
| Entropy (linear) x Baseline | -0.000 | 0.001 | -0.003 – 0.003 | -0.096 | 0.9239 |
| Entropy(quadratic) x Baseline | -0.026 | 0.010 | -0.046 – -0.005 | -2.473 | 0.0134 |
| Participant | 0.011 | 0.007 | -0.002 – 0.025 | 1.644 | 0.1002 |
| Observations | 9831 | | | | |
| R^2^ / adjusted R^2^ | 0.005 / 0.004 | | | | |

**Supplementary file 9. Estimates and statistics of the model predicting post-stimulus low-frequency phase coherence.**
