# Supplementary material for: Local cortical desynchronization and pupil-linked arousal differentially shape brain states for optimal sensory performance
Source: eLife. 2019 Dec 10;8:e51501. doi: 10.7554/eLife.51501 (PMC6946578; doi:10.7554/eLife.51501)
Supplement: Supplementary file 10. — The table shows model coefficients, standard errors, effect size estimates as well as goodness of fit statistics for the model reported in results and discussion sections. [file elife-51501-supp10.docx]

| **Table S10: Brain-behavior model predicting decisions (high vs. low)** | | | | | |
| --- | --- | --- | --- | --- | --- |
|  | **Decision** | | | | |
| *Predictors* | *Log-Odds* | *std. Error* | *CI* | *z-value* | *p* |
| Intercept | -0.057 | 0.148 | -0.347 – 0.233 | -0.384 | 0.701 |
| Pitch | 3.776 | 0.216 | 3.354 – 4.199 | 17.513 | <0.001 |
| Entropy (linear) | 0.024 | 0.030 | -0.034 – 0.082 | 0.801 | 0.423 |
| **Entropy (quadratic)** | **-0.059** | **0.026** | **-0.109 – -0.009** | **-2.311** | **0.021** |
| Baseline Entropy | 0.002 | 0.035 | -0.067 – 0.070 | 0.051 | 0.959 |
| **Pupil size (linear)** | **0.115** | **0.028** | **0.059 – 0.170** | **4.049** | **<0.001** |
| Pupil size (quadratic) | 0.028 | 0.016 | -0.004 – 0.060 | 1.722 | 0.085 |
| Trial number | 0.073 | 0.027 | 0.020 – 0.126 | 2.679 | 0.007 |
| Pitch x Entropy (linear) | -0.126 | 0.070 | -0.263 – 0.012 | -1.788 | 0.074 |
| Pitch x Entropy (quadratic) | -0.047 | 0.058 | -0.161 – 0.066 | -0.817 | 0.414 |
| Pitch x Baseline Entropy | -0.022 | 0.085 | -0.190 – 0.145 | -0.262 | 0.793 |
| Entropy (linear) x Baseline Entropy | 0.090 | 0.029 | 0.033 – 0.147 | 3.104 | 0.002 |
| Entropy (quadratic) x Baseline Entropy | 0.007 | 0.019 | -0.030 – 0.045 | 0.388 | 0.698 |
| **Pitch x Pupil size (linear)** | **-0.232** | **0.068** | **-0.365 – -0.098** | **-3.405** | **0.001** |
| **Pitch x Pupil size (quadratic)** | **-0.153** | **0.035** | **-0.222 – -0.085** | **-4.371** | **<0.001** |
| Pitch x Entropy (linear) x Baseline Entropy | 0.058 | 0.070 | -0.079 – 0.195 | 0.825 | 0.409 |
| Pitch x Entropy (quadratic) x Baseline Entropy | -0.002 | 0.045 | -0.091 – 0.087 | -0.040 | 0.968 |
| **Random Effects** | | | | | |
| σ^2^ | 3.29 | | | | |
| τ_00_ _id_ | 0.49 | | | | |
| τ_11_ _id.semitones_ | 0.86 | | | | |
| ρ_01_ _id_ | -0.31 | | | | |
| Observations | 9831 | | | | |
| Marginal R^2^ / Conditional R^2^ | 0.542 / 0.632 | | | | |

**Supplementary file 10. Estimates and statistics of the model predicting decisions (high vs. low).**
