# Supplementary material for: Local cortical desynchronization and pupil-linked arousal differentially shape brain states for optimal sensory performance
Source: eLife. 2019 Dec 10;8:e51501. doi: 10.7554/eLife.51501 (PMC6946578; doi:10.7554/eLife.51501)
Supplement: Supplementary file 11. — The table shows model coefficients, standard errors, effect size estimates as well as goodness of fit statistics for the model reported in results and discussion sections. [file elife-51501-supp11.docx]

| **Table S11: Brain-behavior model predicting decisions (high vs. low)** | | | | | | |
| --- | --- | --- | --- | --- | --- | --- |
|  | **Decision** | | | | |  |
| *Predictors* | *Log-Odds* | *std. Error* | *CI* | *z-value* | *p* |  |
| Intercept | -0.131 | 0.146 | -0.418 – 0.155 | -0.898 | 0.369 |  |
| Pitch | 3.757 | 0.213 | 3.339 – 4.175 | 17.618 | <0.001 |  |
| Visual cortex entropy (linear) | 0.006 | 0.034 | -0.060 – 0.072 | 0.185 | 0.853 |  |
| **Visual cortex entropy (quadratic)** | **0.009** | **0.021** | **-0.032 – 0.050** | **0.435** | **0.663** |  |
| Baseline Entropy | -0.022 | 0.034 | -0.090 – 0.045 | -0.647 | 0.518 |  |
| **Pupil size (linear)** | **0.112** | **0.029** | **0.056 – 0.168** | **3.925** | **<0.001** |  |
| Pupil size (quadratic) | 0.026 | 0.016 | -0.006 – 0.058 | 1.607 | 0.108 |  |
| Trial number | 0.072 | 0.027 | 0.019 – 0.126 | 2.667 | 0.008 |  |
| Pitch x Entropy (linear) | -0.186 | 0.081 | -0.344 – -0.027 | -2.300 | 0.022 |  |
| Pitch x Entropy (quadratic) | 0.044 | 0.054 | -0.063 – 0.150 | 0.805 | 0.421 |  |
| Pitch x Baseline Entropy | -0.036 | 0.081 | -0.194 – 0.122 | -0.443 | 0.658 |  |
| Visual entropy (linear) x Baseline Entropy | 0.065 | 0.029 | 0.009 – 0.121 | 2.258 | 0.024 |  |
| Visual Entropy (quadratic) x Baseline Entropy | -0.010 | 0.014 | -0.037 – 0.018 | -0.700 | 0.484 |  |
| **Pitch x Pupil size (linear)** | **-0.249** | **0.069** | **-0.384 – -0.114** | **-3.615** | **<0.001** |  |
| **Pitch x Pupil size(quadratic)** | **-0.155** | **0.035** | **-0.224 – -0.086** | **-4.396** | **<0.001** |  |
| Pitch x Visual Entropy (linear) x Baseline Entropy | -0.135 | 0.067 | -0.266 – -0.004 | -2.027 | 0.043 |  |
| Pitch x Visual Entropy (quadratic) x Baseline Entropy | 0.052 | 0.026 | 0.002 – 0.102 | 2.030 | 0.042 |  |
| **Random Effects** | | | | | | |
| σ^2^ | 3.29 | | | | |  |
| τ_00_ _id_ | 0.48 | | | | |  |
| τ_11_ _id.semitones_ | 0.86 | | | | |  |
| ρ_01_ _id_ | -0.32 | | | | |  |
| Observations | 9831 | | | | |  |
| Marginal R^2^ / Conditional R^2^ | 0.544 / 0.633 | | | | |  |

**Supplementary file 11. Estimates and statistics of the model predicting decisions based on visual cortex entropy.**
