# Supplementary material for: Local cortical desynchronization and pupil-linked arousal differentially shape brain states for optimal sensory performance
Source: eLife. 2019 Dec 10;8:e51501. doi: 10.7554/eLife.51501 (PMC6946578; doi:10.7554/eLife.51501)
Supplement: Supplementary file 12. — The table shows model coefficients, standard errors, effect size estimates as well as goodness of fit statistics for the model reported in results and discussion sections. [file elife-51501-supp12.docx]

| **Table S12: Brain-behavior model predicting response speed** | | | | | |
| --- | --- | --- | --- | --- | --- |
|  | **RS** | | | | |
| *Predictors* | *Estimates* | *std. Error* | *CI* | *t-value* | *p* |
| Intercept | 1.632 | 0.040 | 1.566 – 1.698 | 40.584 | <0.001 |
| Task ease | -0.103 | 0.004 | -0.109 – -0.096 | -25.930 | <0.001 |
| Entropy (linear) | -0.007 | 0.004 | -0.014 – 0.001 | -1.511 | 0.1308 |
| **Entropy (quadratic)** | **-0.012** | **0.004** | **-0.018 – -0.005** | **-3.079** | **0.0021** |
| Baseline Entropy | 0.003 | 0.005 | -0.006 – 0.012 | 0.545 | 0.5859 |
| Pupil size (linear) | -0.007 | 0.004 | -0.015 – -0.000 | -1.722 | 0.0850 |
| Pupil size (quadratic) | -0.004 | 0.003 | -0.009 – 0.000 | -1.641 | 0.1007 |
| Entropy (linear) x Entropy baseline | 0.001 | 0.004 | -0.007 – 0.008 | 0.134 | 0.8930 |
| Entropy (quadratic) x Entropy baseline | -0.003 | 0.003 | -0.008 – 0.002 | -1.088 | 0.2766 |
| **Random Effects** | | | | | |
| σ^2^ | 0.16 | | | | |
| τ_00_ _id_ | 0.04 | | | | |
| Observations | 9655 | | | | |
| Marginal R^2^ / Conditional R^2^ | 0.056 / 0.228 | | | | |

**Supplementary file 12. Estimates and statistics of the model predicting response speed.**
